# Multicentric evaluation of sensitivity of eight commercial anti-SARS-CoV-2 antibody assays and their correlation to virus neutralization titers in seropositive subjects

**DOI:** 10.1038/s41598-024-51968-x

**Published:** 2024-01-16

**Authors:** Miroslav Fajfr, Petr Pajer, Daniel Ruzek, Radek Sleha, Sylva Janovska, Milos Bohonek, Hana Kabickova, Pavla Kubicková, Michal Stefanik, Petra Strakova, Pavel Bostik

**Affiliations:** 1 Institute of Clinical Microbiology, University Hospital in Hradec Kralove, Sokolska 581, Hradec Kralove, 50005 Czech Republic; 2 Faculty of Medicine in Hradec Kralove, Charles University, Hradec Kralove, Czech Republic; 3 Military Health Institute, Prague, Czech Republic; 4 Department of Virology, Veterinary Research Institute, Brno, Czech Republic; 5 Institute of Parasitology, Biology Centre of the Czech Academy of Sciences, Ceske Budejovice, Czech Republic; 6 Department of Experimental Biology, Faculty of Science, Masaryk University, Brno, Czech Republic; 7 Department of Epidemiology, Military Faculty of Medicine, University of Defence, Hradec Kralove, Czech Republic; 8 Department of Hematology and Blood Transfusion, Military University Hospital Prague, Praha, Czech Republic; 9 Faculty of Biomedical Engineering, Czech Technical University in Prague, Prague, Czech Republic

**Keywords:** Clinical microbiology, Infectious-disease diagnostics, SARS-CoV-2

## Abstract

Diagnosis of SARS-CoV-2 virus is mainly based on direct detection. Determination of specific antibodies has been used mostly for epidemiological reasons. However, select immunoassays showed good correlation to plaque reduction virus neutralization test (PRNT) in smaller patient cohorts, which suggests their potential as predictors of virus neutralization titer. A total of 3,699 samples from Covid-19 patients were included in the multicentric study performed in the Czech Republic. Anti-SARS-CoV-2 antibody levels were evaluated by 8 commercial antibody assays. Simultaneously, PRNT evaluations were performed with the SARS-CoV-2 B.1.258 variant. All immunoassays showed an overall high true positive diagnostic value ranging from 79.17 to 98.04%. Several commercial EIA methods showed highly positive correlation between the assay results and PRNT levels, e.g., Liaison CoV-2 TrimericS IgG DiaSorin (Spearman r = 0.8833; Architect SASRS-CoV-2 IgG Abbott (r = 0.7298); NovaLisa SARS-CoV-2 IgG NovaTec (r = 0.7103) and Anti-SARS-CoV-2 ELISA IgG Euroimmun (r = 0.7094). While this correlation was less positive for other assays, those, conversely, presented higher true positive values. For most immunoassays, the positive percent agreement of the results was ≥ 95% in sera exhibiting PRNT levels of 1:80 and higher. The assays tested have shown variable correlation to PRNT. Those possessing high positive predictive values serve well as qualitative tests, while others can be utilised as quantitative tests highly predictive of neutralization antibody levels.

## Introduction

Coronaviruses are known human respiratory pathogens. Several human coronaviruses are causative agents of seasonal mild influenza-like infections, e.g. alphacoronaviruses HCoV-229E or HCoV-NL63 and betacoronaviruses HCoV-OC43 and HCoV-HKU1. Few other beta-coronaviruses were shown previously to cause severe respiratory infections—SARS-CoV or MERS-CoV. In 2019, a new human coronavirus emerged and caused a global pandemic. This new coronavirus has been named SARS-CoV-2 due to its genetical and symptomatical similarity to SARS-CoV-1. The new SARS-CoV-2 causes Coronavirus diseases 2019 (COVID-19), which is characterized by a varying severity of disease, ranging from mild respiratory infections to the severe respiratory distress syndrome and respiratory failure. The main diagnostic tools used for the diagnosis of COVID-19 have been PCR assays and SARS-CoV-2 specific antigen detection tests. Serological assays (the detection of virus specific antibodies) have been mostly used as auxiliary methods, in the Czech Republic often only for screening of convalescent plasma donors or as an effective epidemiological tool for identifying past infections, but rarely for COVID-19 diagnostic purposes.

A broad spectrum of serologic methods for establishing specific SARS CoV-2 antibodies have been used, ranging from rapid "first-line" diagnosis by immunochromatographic assays to the most accurate virus neutralization assay. The rapid tests showed relatively lower sensitivity in a comprehensive metadata analysis by the Cochrane Institute^1^, but have been widely used as the first-line tool due to its cost and simple manipulation. The EIA/CMIA/CLIA tests are used in clinical laboratories and, currently, wide variety of commercial tests characterised by varying efficacies are available. Several studies have compared these tests with each other and with virus neutralization tests and showed, that the sensitivities and degrees of correlation to the virus neutralization test results are relatively variable^2–7^. One potential reason for these discrepancies could be the variability of targets utilised in the individual tests, with the most used ones being the nucleocapsid antigen, the S1 antigen, or the RBD domain of the S1 antigen. During the immune response in COVID-19, the development of antibodies against these individual targets shows different dynamics^3,8,9^. The level of neutralizing antibodies appears to be a reliable marker for predicting immune protection from symptomatic SARS-CoV-2 infection, and therefore knowledge of their level could lead to better implementation of the serological method in SARS-CoV-2 diagnostics^10^. Thus, the purpose of this study was to test the most used commercially available SARS-CoV-2 antibody assays on a large cohort of patient sera and evaluate their predictive values for the virus neutralization potential of the detected antibodies.

## Materials and methods

### Sample collection

Patient sera or plasma were collected from three large hospitals in the Czech Republic during the period of April 2020–January 2021. A total of 3,699 samples obtained from patients at various time periods after SARS-CoV-2 infection (from acute samples to convalescent samples at 12 months post-acute infection) were included in the study. Positivity was confirmed in all patients by the PCR test from respiratory samples. All data were anonymized and subsequent processing of the data was performed strictly according to the Helsinki protocol^11^. The study was approved by the Institutional Ethics Committee of Faculty Hospital in Hradec Kralove (reference number 202101I33P approved on December 21, 2020). Informed consent was obtained from all subjects and/or their legal guardians. All the samples were evaluated by either one or more enzyme-linked immunosorbent assays/chemiluminescent immunoassays/chemiluminiscent microparticle immunoassays (EIA/CLIA/CMIA) and the plaque reduction virus neutralization test (PRNT). Because the analysis focused on a comparison between commercial diagnostic immunoassays and virus neutralization test regardless of clinical manifestations, the stage of COVID-19 was not considered.

### Enzyme immune assays

In total, eight different EIA, CMIA, or eCLIA (electro chemiluminiscent immunoassay) tests were used in the study. These included: Anti-SARS-CoV-2 ELISA IgG (Euroimmun, Lübeck, Germany), Elecsys Anti-SARS-CoV-2 S, Cobas (Roche, Mannheim, Germany) in both quantitative and semi-quantitative variants, Maglumi SARS-CoV-2 neutralizing antibody (CLIA) (Snibe Diagnostic, Shenzhen, China), SARS-CoV-2 NP IgG ELISA Kit (ImmunoDiagnostics, Shenzhen, China), Architect SASRS-CoV-2 IgG (Abbott, Sligo, Ireland), NovaLisa SARS-CoV-2 IgG (NovaTec Immunodiagnostica, Dietzenbach, Germany) and Liaison SARS-CoV-2 TrimericS IgG (DiaSorin, Stillwater, USA). Table 1 shows a comprehensive summary for each method and batch numbers of each test are listed in Supplementary Table 1.

**Table 1:** Basic characteristics of EIA/CLIA/CMIA methods used in the study.

Method	Manufacturer	Assay type	Target	Result value	Borderline value	Manufact. sensitivity	Manufact. Specificity (%)	Number of samples in tested
Anti-SARS-CoV-2 ELISA IgG	EUROIMMUN	ELISA	S antigen (S1 subunit)	Semiquant (S/CO)	0.8–1.1	93.2%*#	99.8	1672
SARS-CoV-2 NP IgG ELISA kit	ImmunoDiagnostics	ELISA	NC antigen	Semiquant (S/CO)	0.9–1.1	92.5%	93.33	369
NovaLisa ® SARS-CoV-2 IgG	NovaTec Immunodiagnostica	ELISA	NC antigen	Quantitative (NTU)	9–11	100.0%*#	99.24	101
Elecsys Anti-SARS-CoV-2 S, Cobas	ROCHE	ECLIA	S antigen (RBD domain)	Quantitative (U/ml)	0.8	96.6%	99.98	594
Elecsys Anti-SARS-CoV-2, Cobas	ROCHE	ECLIA	NC antigen	Semiquant (S/CO)	1.0			148
Maglumi SARS-CoV-2	Snibe Diagnostic	CLIA	recombinant S (RBD domain)/NC antigen	Quantitative (AU/ml)	1.0	99.6%*	100*	487
Architect SARS-CoV-2 IgG	Abbott	CMIA	NC antigen	Semiquant (S/CO)	1.4	96.77%*°	99.63	230
Liaison SARS-CoV-2 TrimericS IgG	DiaSorin	CLIA	S antigen	Quantitative (AU/ml)	13.0	98.7%*°	99.5	98

Notes—S—spike, NC—nucleocapsid,

*Clinical sensitivity and specificity.

°Sensitivity over 14 days after PCR test/symptoms onset.

^#^Sensitivity over 21 days after PCR test/symptoms onset.

### Plaque reduction virus neutralization test (PRNT)

The clinical isolate of the original SARS-CoV-2 virus (strain SARS-CoV-2 variant B.1.258, isolated from a clinical sample at the National Institute of Public Health, Prague) was used in this study. In addition, 88 samples were analysed using variant Alpha (B.1.1.7). Virus stocks were prepared by infecting susceptible Vero cells CCL81 line in the University Hospital Hradec Kralove laboratory (HK) and Vero cells E6 line in Prague Military Health Institute—Centre for Biological Defence Techonin (PG) and Brno Veterinary Research Institute (BR) laboratories. All participating laboratories are certified at least as the BSL 3 level. Viral titers were determined using the 50% tissue culture infectious doses assay (TCID_50_), or plaque assay, as described previously^12^. Briefly, serial two-fold dilutions (starting at 1:20) of serum samples in DMEM were prepared in 96-well plates seeded with the Vero cells. The virus neutralizing antibody titer (VNT) was determined as the highest serum dilution that prevents the cytopathic effect (CPE) in duplicate wells. The titer of the virus was calculated according to the methods of Spearman and Karber and expressed as log_10_ TCID_50_/ml. The virus neutralization assay was performed according to the protocol described in Manenti et al.^13^. The neutralizing antibody titer was determined as the highest serum dilution preventing CPE in duplicate wells. The correlation tests of PRNT were performed among all participating laboratories to ensure, that the results were directly comparable. All three laboratories performed two runs of a blinded comparative test provided by the Czech national reference laboratory. The results showed variations only within one dilution of samples.

### Statistics

The GraphPad Prism 9 software (version 9.20, GraphPad Software Inc., San Diego, CA USA) was utilised for graphical outputs and basic statistical evaluation. The normality evaluation was performed using the Anderson–Darling test and Shapiro–Wilk test. For the correlation of the EIA methods with the virus neutralization test, the nonparametric Spearman's rank correlation coefficient test with a 95% confidence interval was used.

## Results

SARS-CoV-2 antibodies from 3699 individual serum samples were quantitated by one or more commercially available COVID-19 antibody assays and simultaneously tested for their PRNT activity. The goal was to identify the correlations between the results of the specific antibody levels detected by the immunoassay and the PRNT levels. Those immunoassays, which show highly significant positive correlation results then can be recommended to serve as the correlate value of the virus neutralization capacity of the antibodies detected.

**Figure 1:**
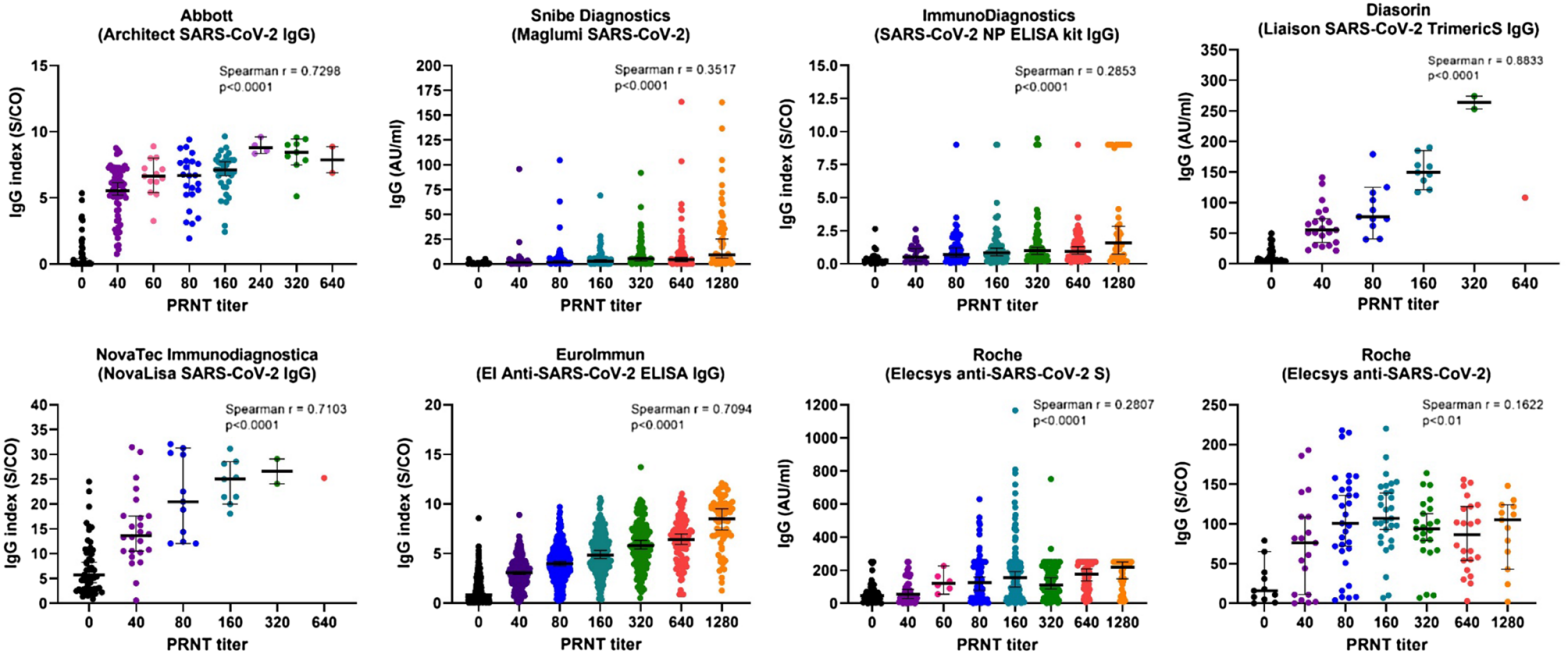
The correlation of the anti SARS-CoV-2 IgG values obtained by the individual immunoassays and TCID_50_ virus neutralization titers in serum samples. The detected antibody levels (expressed either as the IgG index or in AU/ml, depending on the assay) are correlated to the PRNT levels using the nonparametric Spearman´s rank correlation coefficient test.

Thus, an evaluation of the correlation between the levels of specific IgG antibodies (detected by EIA) and the titers of virus neutralization antibodies was performed using the Spearman correlation test (Fig. 1).

Due to the fact, that the PRNT tests were initially calibrated among all the laboratories, it was possible to include samples tested in all laboratories with the particular EIA in the cumulative analysis. The results showed that several EIA methods, i.e. Liason SARS-CoV-2 Trimeric IgG (r = 0.8833), Architect SARS-CoV-2 IgG (r = 0.7298); NovaLisa SARS-CoV-2 IgG (r = 0.7103) and Euroimmun SARS-CoV-2 IgG (r = 0.7094) exhibited a highly significant correlation between the IgG antibody index and PRNT (p < 0.001). The remaining four commercial assays showed significantly lower positive correlations between the IgG antibody indices and PRNT levels (r = 0.1622 to r = 0.3662). This indicates that the former set of assays with the high Spearman R values represents more reliable predictors of virus neutralization serum levels and can be utilized as such.

**Figure 2:**
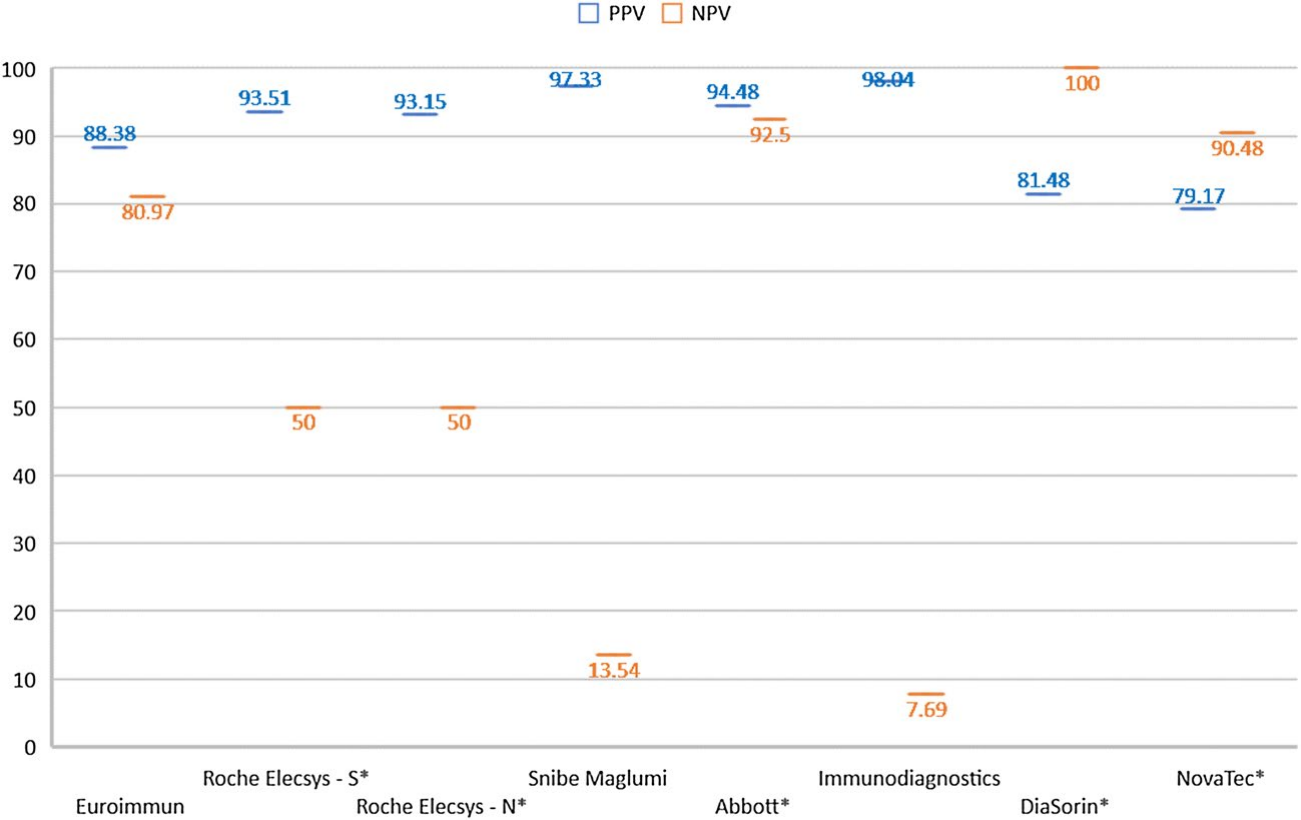
The overall PPV—positive predictive value (blue color) and NPV—negative predictive value (red color) of the immunoassay results compared to the overall PRNT results. * indicates numbers of negative samples < 100 used for NPV calculation.

Further analysis was performed to evaluate the overall accuracy of the individual immunoassay with PRNT. To achieve that, the statistical analysis of the overall positive predictive value (PPV, calculated as true positivity against true positivity + false positivity) and negative predictive value (NPV, calculated as true negativity against true negativity + false negativity) independently to immunoassay value or PRNT titre was performed (Fig. 2).

The PPV data showed a very good correlation (93.15–98.04%) for the assays from Roche (with both the anti-S and anti-N variants), Snibe Diagnostic, Abbott, and Immunodiagnostics. The remaining three assays showed somewhat lower, but still relatively good correlation—79.17–88.38%. For the the NPA, the data showed highly reliable correlation (from 90.48 to 100.00%) in three assays, i.e. Abbott, DiaSorin and NovaTec, and the still acceptable result of 80.97% for the Euroimmun assay. On the contrary, the results for the Snibe Diagnostic and Immunodiagnostics assays showed poorer correlation in true negativity (13.54% and 7.69% NPV, respectively). The Roche test ranked in-between with only 50.0% correlation of the negativity with the PRNT. However, only smaller numbers of negative results were included in the NPV data evaluation, which may bias the results for these assays. Taken together the assays from Abbott, DiaSorin, NovaTec, and Euroimmun provided the best results for both the PPV and NPV correlation.

To further analyse in more detail, how the positive results from the individual assays (PPA) are dependent on the levels of PRNT titers in the individual samples, the cohort was stratified into subgroups with the defined minimum PRNT titer and for each of these subgroups the PPA was calculated (Table 2).

**Table 2:** The positive agreement correlation of the general positivity (PPV in %) of the individual antibody assays at the different PRNT levels.

PRNT titer*	Euroimmun	Roche Elecsys S	Roche Elecsys N	Snibe Diagnostic	Abbott	Immuno Diagnostics	DiaSorin	NovaTec
40 +	94.34%	99.27%	99.27%	81.43%	98.09%	44.12%	100.00%	80.85%
80 +	96.73%	99.60%	100.00%	82.77%	100.00%	45.45%	100.00%	100.00%
160 +	98.16%	99.48%	100.00%	84.10%	100.00%	47.22%	100.00%	100.00%
320 +	98.60%	100.00%	100.00%	85.20%	100.00%	50.85%	100.00%	100.00%
640 +	98.38%	100.00%	100.00%	84.21%	100.00%	52.17%	100.00%	100.00%
1280 +	100.00%	100.00%	100.00%	88.68%	NA	61.90%	NA	NA

*The numbers in each row indicate the minimal PRNT positivity level of the samples included in the analysis of the PPV. Thus, “40 + ” denotes all the samples, where the detected PRNT was 1:40 or higher.

From this evaluation, an excellent correlation (98.60–100.00%) was observed for higher PRNT titers (greater than 1:320) in all but two assays. The correlation of the Snibe Diagnostics assay ranged from 81.43 to 88.68% for all positive PRNT titers. The ImmunoDiagnostics assay results showed insufficient correlation for all PRNT titers measured, ranging from 44.12% for titers of 1:40 and higher to 61.90% for titers of 1:1,280 and higher. Samples with PRNT titers below 1:320 showed some minor decreases in the correlation to the EIA positivity (PPV) in the remaining tests. Thus, in the samples exhibiting titers 1:40 and higher the correlation decreased to 80.85% in the NovaTec assay and to 94.34% for Euroimmun. But the remaining assays (Roche, Abbott and DiaSorin) showed still PPA levels close to 100%, even when the samples with low PRNT levels were included (99.27%, 98.09% and 100.00%, respectively).

**Figure 3:**
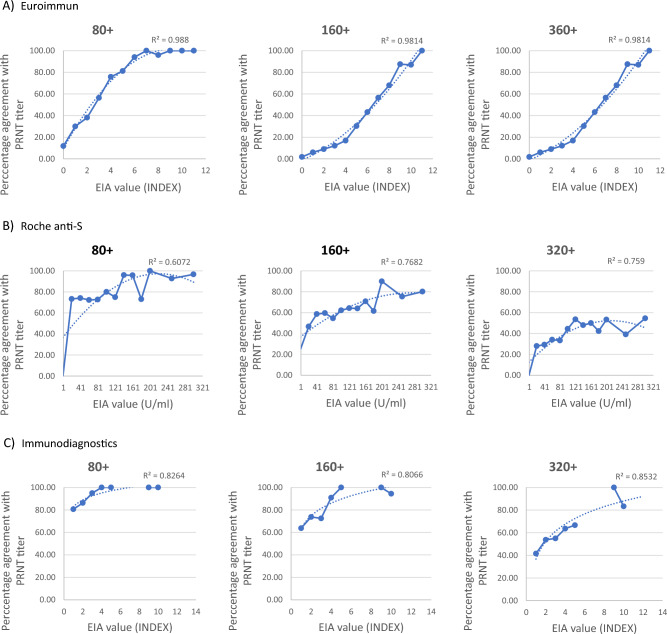
A comparison of the performance of detection scale of the select antibody assays with the PRNT test. ROC curves for values obtained from the individual assays were calculated for samples with PRNT titers ≥ 1:80, ≥ 1:160 and ≥ 1:320. The panels show the ratio of the assay results in % for the individual assay IgG index result values (x-axis) in (**A**) Euroimmun assay, (**B**) Roche anti-S assay and (**C**) Immunodiagnostics assay for the samples with the PRNT titer higher than indicated in the individual charts. The Coefficients of determination R^2^ were calculated for each of the results (MS Excell® statistics).

In summary, only four assays showed sufficient correlation between the value of EIA and PRNT titer—DiaSorin, Abbott, Euroimmun, and NovaTec platforms. The same assays showed balanced overall true-positive and true-negative values. The Roche, Snibe Diagnostic, and Immunodiagnostics platforms showed better overall true-positive values, but also had high levels of false negatives. We found only little differences between assays using nucleocapsid or subunits of S antigens as targets. Because no definitive PRNT titer value was established to date as beeing clearly protective, the PRNT titer considered as sufficient for convalescent plasma donors (1:160) has been used previously as the significant titer value^14^. To further analyse the performance of the assays used to analyse large numbers of samples (Euroimmun, Roche, and Immunodiagnostics), the Regression curves (R^2^C) for samples with PRNT titers of 1:80, 1:160, and 1:320 were calculated (Fig. 3). The constructed R^2^C curves presented percentage agreement of the EIA values with the samples with the indicated PRNT titers. The results show that all three analysed assays reach 90% confidence for the analysis of cohorts showing PRNT titers higher than 1:80. In the remaining two cohorts with PRNT titers ≥ 1:160 and ≥ 1:320, respectively, only Euroimmun and ImmunoDiagnostics assays were able to reach 90% probability. The 90% confidence in reaching the individual PRNT titers is used as a threshold which indicates whether the particular EIA assay value reflects the PRNT titer with a sufficient probability.

The evaluation of potential effect of the individual virus variants on the neutralizing antibody titers was performed by a comparison the PRNT titers in 88 sera samples using two SARS-CoV-2 variants—B.1.258 and Alpha. The results showed a correlation of the PRNT titers in 22.4% of positive samples and in 93.3% of negative samples. Most of the positive samples (60.4%) showed lower PRNT titers when the Alpha virus variant was used (Supplementary Table 2).

## Discussion

Earlier studies compared the available SARS-CoV-2 antibody assays and correlated their clinical sensitivity and specificity as a function of time after infection. According to these studies, substantial differences in sensitivities and specificities of these assays were found in the individual analyses^2,4,5,15,16^. Studies conducted later, similarly to the study presented herein, correlated the results to the levels obtained by virus neutralization assays to provide more accurate sensitivity/specificity results and correlate the results to the neutralization antibody levels^3,5–7^.

A detailed knowledge of neutralizing antibody levels can be used as a reliable tool to predict the clinical course of COVID -19 and immune protection against SARS-CoV-2 infection^17^. According to the earlier studies, neutralizing antibodies persist for many months^18,19^. It would be rather difficult, or even impossible to routinely test the virus neutralization antibody levels due to the necessary safety precautions for work with the live virus. Therefore, establishing other ways of measuring neutralization antibody levels, or their correlates are needed. One of these ways is to use an EIA platform exhibiting significant correlation of the results to the virus neutralization antibody titers. A previous study has shown that positive linear regression with the PRNT is statistically lower for ELISA platforms using nucleocapsid proteins (r^2^ = 0.09) than for platforms using S1 or RBD (r^2^ = 0.35 and r^2^ = 0.38, respectively) as targets^3^. However, in our study, we found no evidence of the quality and fidelity of the results depending only on the assay target.

Authors of the study mentioned above^3^ compared two automated serologic assays (Abbott and Ortho) and three in-house ELISA assays (against S1, RBD, and NC targets) with the neutralizing activity levels established using pseudotyped viruses. Their results showed a high degree of correlation for both commercial assays (Spearman r = 0.75 for Ortho and r = 0.72 for Abbott assay) and for the in-house ELISAs (r = 0.65–0.69). Similar results were also published by Patel et al.^5^. Good correlations to the AUC values of the neutralizing antibody titers were found for the Euroimmun and Epitope Diagnostics assays (Spearman r = 0.81 and 0.74, respectively) and poorer correlation for the Roche assay (r = 0.40). These data agree with our results, where for the DiaSorin assay Spearman score of r = 0.8833 was calculated and three other assays (both automated and manual—Abbott, Euroimmun and NovaTec) showed correlation coefficients above 0.7. Furthermore, poorer correlation values were found for three assays (Roche and Snibe) (r = 0.1622 to r = 0.3662). Analysis of our data showed that the degree of true positivity was above 80% for all the assays tested (except NovaTec with 79.17%), but the degree of true negativity was above 80% for only 4 assays. These results are also in agreement with the study mentioned above^5^.

It has been repeatedly discussed whether the development of SARS-CoV-2 variants and their introduction on the circulation could affect the correlation of EIA antibody measurements to the neutralization capacity of the antibodies. Several published studies showed a relatively high correlation between the EIA and PRNT results across several SARS-CoV-2 circulating variants. Thus, for clades 19, Alpha, Beta, Gamma and Delta, the correlation coefficient varied between r = 0.71–0.79 for using the iFlash-2019-nCoV Nab assay (Yhlo Biotechnologies, China) or between r = 0.83–0.96 using the Vidas SARS-CoV-2 IgG assay (bioMérieux, France)^20^. Very similar results were published from Canada, where the Abbott SARS-CoV-2 IgG II Quant assay (Abbott, USA) showed the correlation coefficient in the range of r = 0.70–0.85 for the variants wild-type, Alpha, Beta, Delta and Gamma^21^. Other studies showed discrepancies of the neutralizing antibodies titers (in the majority of studies generated by different SARS-CoV-2 vaccines) depending on SARS-CoV-2 variants. These studies showed significantly lower neutralizing antibodies titres against the Omicron variant compared to the wild type virus^22^, Omicron BA.1 compared to the Delta variant^23^, or decreases in medians of the neutralizing antibody titers median and their prevalence in the Omicron, Delta and Gamma variants^24^. In our work, similar results were seen when comparing B.1.158 and Alpha variants. In addition, Lee and co-workers also showed different dynamics of the PRNT titer waning for different SARS-CoV-2 variants^25^. All limitations of the detected PRNT levels in correlation to the different SARS-CoV-2 variants must be taken into account in correlation studies. But despite the results from our study can be interpolated for other virus variants and serve as a valuable resource obtained on a large cohort of samples, albeit with the previously circulating virus variants.

The results of our study indicate that immunoassays can be differentiated into two main groups based on the expected output. The first set contains assays exhibiting high true positivity, low true negativity, and a poorer correlation between the EIA value and the PRNT titer. These assays are useful as highly reliable qualitative tests with a high positive predictive value, but unsuitable for predicting the PRNT titer. This applies to the assays from Roche, Snibe Diagnostics, and ImmunoDiagnostics. The second set of assays shows a balanced true positivity and true negativity, and a sufficient correlation to the PRNT titer. This set includes the DiaSorin, Abbott, Euroimmun and NovaTec assays. For these tests, the degree of correlation between the EIA value and the PRNT titer can be calculated, which allows for the prediction of the antibody neutralization titer from the EIA value. Thus, these tests can be used for more accurate measurements of the correlates of protection.

## Conclusions

Our results showed very high correlation between the immunoassay value and the titer of the virus neutralization antibodies in several commercial assays. The results also suggest two potentially different application purposes of the individual immunoassays, depending on the quality measured: i.e. as a qualitative test with higher positive predictive value of sample positivity, or as a quantitative test with higher confidence in the correlation of the test values to the neutralization capacity of the individual samples.

### Supplementary Information

Supplementary Table 1.

Supplementary Table 2.

## Data Availability

Data are available on request from Miroslav Fajfr (miroslav.fajfr@fnhk.cz) due to the ethical restrictions.
