# Supplementary material for: Multicentric evaluation of sensitivity of eight commercial anti-SARS-CoV-2 antibody assays and their correlation to virus neutralization titers in seropositive subjects
Source: Sci Rep. 2024 Jan 16;14:1421. doi: 10.1038/s41598-024-51968-x (PMC10792077; doi:10.1038/s41598-024-51968-x)
Supplement: Supplementary file 1 — Supplementary Table 1. [file 41598_2024_51968_MOESM1_ESM.docx]

**Supplementary Table 1.** Batch numbers of the tests utilized in the sudy.

| **Method** | **Manufacturer** | **Assay Batch No** |
| --- | --- | --- |
| Anti-SARS-CoV-2 ELISA IgG | Euroimmun | E200429AG, E201222AC |
| SARS-CoV-2 NP IgG ELISA kit | ImmunoDiagnostics | A31003 |
| NovaLisa ® SARS-CoV-2 IgG | NovaTec Immunodiagnostica | 3014118 |
| Elecsys Anti-SARS-CoV-2 S, Cobas | ROCHE | 647389 |
| Elecsys Anti-SARS-CoV-2, Cobas | ROCHE | 54549201 |
| Maglumi SARS-CoV-2 | Snibe Diagnostic | 30020060901 |
| Architect SARS-CoV-2 IgG | Abbott | 44367FN00 |
| Liaison SARS-CoV-2 TrimericS IgG | DiaSorin | 136290c |
