# Supplementary material for: Multicentric evaluation of sensitivity of eight commercial anti-SARS-CoV-2 antibody assays and their correlation to virus neutralization titers in seropositive subjects
Source: Sci Rep. 2024 Jan 16;14:1421. doi: 10.1038/s41598-024-51968-x (PMC10792077; doi:10.1038/s41598-024-51968-x)
Supplement: Supplementary file 2 — Supplementary Table 2. [file 41598_2024_51968_MOESM2_ESM.docx]

**Supplementary Table 2.** Comparison of PRNT titers in selected sera using B.1.258 and B.1.1.7 SARS-CoV-2 variants.

| **B.1.258 positive sera** | |  |  | **B.1.258 negative sera** | |  |
| --- | --- | --- | --- | --- | --- | --- |
| **B.1.258** | **B.1.1.7** | **comparison*** |  | **B.1.258** | **B.1.1.7** | **comparison*** |
| **160** | **160** | **E** |  | **0** | 0 | E |
| **160** | **40** | **D** |  | **0** | 0 | E |
| **320** | **160** | **D** |  | **0** | 0 | E |
| **320** | **160** | **D** |  | **0** | 0 | E |
| **320** | **160** | **D** |  | **0** | 0 | E |
| **320** | **0** | **D** |  | **0** | 0 | E |
| **320** | **160** | **D** |  | **0** | 0 | E |
| **320** | **320** | **E** |  | **0** | 0 | E |
| **320** | **320** | **E** |  | **0** | 0 | E |
| **640** | **160** | **D** |  | **0** | 0 | E |
| **640** | **40** | **D** |  | **0** | 0 | E |
| **640** | **1280** | **I** |  | **0** | 0 | E |
| **640** | **320** | **D** |  | **0** | 0 | E |
| **640** | **320** | **D** |  | **0** | 0 | E |
| **640** | **80** | **D** |  | **0** | 0 | E |
| **640** | **160** | **D** |  | **0** | 0 | E |
| **1280** | **160** | **D** |  | **0** | 0 | E |
| **1280** | **160** | **D** |  | **0** | 0 | E |
| **1280** | **1280** | **E** |  | **0** | 0 | E |
| **1280** | **1280** | **E** |  | **0** | 0 | E |
| **1280** | **1280** | **E** |  | **0** | 0 | E |
| **1280** | **320** | **D** |  | **0** | 0 | E |
| **40** | **160** | **I** |  | **0** | 80 | I |
| **80** | **0** | **D** |  | **0** | 0 | E |
| **160** | **80** | **D** |  | **0** | 40 | I |
| **160** | **40** | **D** |  | **0** | 0 | E |
| **320** | **320** | **E** |  | **0** | 0 | E |
| **320** | **40** | **D** |  | **0** | 0 | E |
| **320** | **160** | **D** |  | **0** | 0 | E |
| **320** | **80** | **D** |  | **0** | 0 | E |
| **640** | **160** | **D** |  |  |  |  |
| **640** | **320** | **D** |  |  |  |  |
| **1280** | **640** | **D** |  |  |  |  |
| **1280** | **80** | **D** |  |  |  |  |
| **1280** | **160** | **D** |  |  |  |  |
| **1280** | **320** | **D** |  |  |  |  |
| **40** | **0** | **D** |  |  |  |  |
| **80** | **0** | **D** |  |  |  |  |
| **80** | **160** | **I** |  |  |  |  |
| **80** | **320** | **I** |  |  |  |  |
| **160** | **80** | **D** |  |  |  |  |
| **160** | **80** | **D** |  |  |  |  |
| **160** | **320** | **I** |  |  |  |  |
| **160** | **80** | **D** |  |  |  |  |
| **160** | **160** | **E** |  |  |  |  |
| **160** | **40** | **D** |  |  |  |  |
| **160** | **160** | **E** |  |  |  |  |
| **160** | **320** | **I** |  |  |  |  |
| **160** | **160** | **E** |  |  |  |  |
| **160** | **640** | **I** |  |  |  |  |
| **320** | **320** | **E** |  |  |  |  |
| **320** | **1280** | **I** |  |  |  |  |
| **320** | **640** | **I** |  |  |  |  |
| **640** | **1280** | **I** |  |  |  |  |
| **160** | **160** | **E** |  |  |  |  |
| **160** | **80** | **D** |  |  |  |  |
| **320** | **160** | **D** |  |  |  |  |
| **320** | **320** | **E** |  |  |  |  |
|  |  |  |  |  |  |  |

* titers identified as E - Equal, I - increased, D- decreased
